# Significance of the Neutrophil-to-Lymphocyte Ratio, Platelet-to-Lymphocyte Ratio, and Preoperative Nutritional Index as Predictors of Morbidity in Patients Who Underwent Liver Resection for Alveolar Echinococcosis

**DOI:** 10.7759/cureus.44842

**Published:** 2023-09-07

**Authors:** Murat Kartal, Nurhak Aksungur, Ercan Korkut, Necip Altundaş, Salih Kara, Gürkan Öztürk

**Affiliations:** 1 General Surgery, Atatürk University Research Hospital, Erzurum, TUR; 2 General Surgery, Atatürk University Faculty of Medicine, Erzurum, TUR

**Keywords:** prognostic nutritional index, neutrophil, morbidity, lymphocyte, hepatic alveolar echinococcosis

## Abstract

Aim: We aimed to evaluate the significance of neutrophil-to-lymphocyte ratio (NLR), platelet-to-lymphocyte ratio (PLR), and preoperative nutritional index (PNI) as predictors of morbidity in patients who underwent liver resection for alveolar echinococcosis.

Material and methods: This single-center study was designed as a retrospective study after obtaining ethical committee approval. The files of patients hospitalized at Ataturk University Faculty of Medicine, Erzurum, Turkey, between 2010 and 2019 and who underwent resection or liver transplantation for liver alveolar cysts were reviewed. Demographic features, laboratory parameters (complete blood count and biochemical parameters), lesion localizations and characteristics, type of surgery, intraoperative and postoperative complications (morbidity), and mortality status were evaluated by scanning patients’ files. Preoperative blood samples were taken the day before the surgery, which is the period farthest from surgical stress, to have more accurate results. By contrast, postoperative blood samples were taken on the first postoperative day when surgical stress was the highest. The differences between the morbidity groups, including NLR, PLR, and PNI, were compared.

Results: Of the 172 patients in the study, 96 (55.8%) were female. The mean age of all patients was 48.51±15.57 (18-90). Perioperative complications were seen in 30 (17.4%) patients, while the morbidity and mortality rates of the study were 28.5% and 19.2%, respectively. Age, gender of patients, and preoperative laboratory parameters, including NLR, PLR, and PNI, did not affect morbidity. However, the presence of perioperative vascular injury (*P*=0.040) and complications (*P*=0.047), low postoperative lymphocyte rates (*P*=0.038), and high postoperative NLR were associated with increased morbidity. In addition, the mortality rate was significantly increased in patients with morbidity (*P*<0.001).

Conclusion: From the results of the present study, it was found that preoperative parameters did not affect morbidity, while increased postoperative NLR levels and decreased lymphocyte rates increased morbidity.

## Introduction

Echinococcosis is a parasitic disease of tapeworms of the *Echinococcus* type. There are four species in the genus *Echinococcus*, namely, *Echinococcus granulosus*, *Echinococcus alveolaris*, *Echinococcus vogeli,* and *Echinococcus oligarthrus* [1]. *E. granulosus* is responsible for the common echinococcosis, and *E. alveolaris* is responsible for 3% of all echinococcal lesions in the liver [2]. Echinococcosis is an endemic disease in Turkey, especially in Eastern Anatolia [3]. The liver disease caused by *E. alveolaris* is a slowly progressive parasitic disease. The incubation period ranges from five to 15 years. The disease is usually fatal due to complications from metastatic lesions or liver failure caused by the primary lesion. The 10-year mortality after initial diagnosis in untreated patients is 95% [4].

The patient's anamnesis, occupation, clinical symptoms, laboratory findings, and imaging of the lesion guide the diagnosis of an alveolar hydatid cyst [5]. Early diagnosis of alveolar hydatid cyst is essential for adequate treatment. There is no specific change in routine laboratory tests. Sedimentation is increased, and there is mild eosinophilia. If splenomegaly is accompanied, the leukocyte and platelet counts decrease. Most patients have hypergammaglobulinemia and increased immunoglobulin E (IgE) levels [6]. Ultrasonography (USG) and computed tomography (CT) take first place as imaging methods in diagnosing the disease. Magnetic resonance imaging (MRI), endoscopic retrograde cholangiopancreatography (ERCP), and percutaneous transhepatic cholangiogram (PTC) are other imaging tools used for diagnosis [7].

Alveolar echinococcosis is a disease that progresses to severe inflammation and can cause local invasion and distant metastasis, just like cancers. Nutritional and immunological status is also affected in this group of patients. Studies have shown that many preoperative prognostic indicators calculated from patient serum samples are associated with cancer symptoms, metastases, outcomes, and prognosis. In many studies, it has been reported that inflammatory factors, such as neutrophil-to-lymphocyte ratio (NLR), platelet-to-lymphocyte ratio (PLR), and preoperative nutritional index (PNI), are influential in the prognosis of various cancer types and various inflammatory diseases [8].

Because there is no study in the literature in which the three parameters were evaluated simultaneously in alveolar echinococcus, this study was planned to fill this gap in the literature. We aimed to evaluate the significance of NLR, PLR, and PNI as predictors of morbidity in patients undergoing liver resection for alveolar echinococcosis.

## Materials and methods

Patient selection

This single-center study was designed as a retrospective study after receiving ethical committee approval from the Non-invasive Clinical Research Ethics Committee of Ataturk University Faculty of Medicine, Erzurum, Turkey (decision no.: 2/date: March 13, 2019). The medical records of 235 patients hospitalized in the Ataturk University Faculty of Medicine Department of General Surgery between 2010 and 2019 and who underwent resection or liver transplantation for liver alveolar cysts were reviewed. Patients younger than 18 years of age; patients with recurrent alveolar echinococcosis; patients treated with R1-R2 resection; patients using drugs that may increase serum neutrophil, thrombocyte, and leukocyte levels; patients with concomitant infection, hematological disease, kidney disease, rheumatological disease, and other types of cancer; and patients with missing file data were not included in the study. After all filtering, 172 patients were eligible for the study.

Searched parameters

The patients’ data were retrieved from the hospital’s computer system and the archives of patients’ medical records. Demographic features, laboratory parameters (complete blood count and biochemical parameters), lesion localizations and characteristics, type of surgery, intraoperative and postoperative complications, and mortality status were evaluated by scanning patients’ files. Preoperative blood samples were taken the day before the surgery, which is the period farthest from surgical stress to have more accurate results. By contrast, postoperative blood samples were taken on the first postoperative day, when surgical stress was the highest. In addition, the other reason for choosing the postoperative first day for postoperative blood samples is that the surgeries performed are major surgeries and to prevent a possible complication, such as intra-abdominal hemorrhage, by seeing the early period blood values.

NLR was calculated from the neutrophil value in the complete blood count divided by the lymphocyte value, and PLR was calculated from the platelet value divided by the lymphocyte value. The PNI is calculated using a simple mathematical formula: PNI = (10 × serum albumin [g/dL]) + (0.005 × lymphocytes/μL). Patients who developed complications within 30 days of surgery or during hospitalization were considered the morbidity-positive group, and those without complications were considered the morbidity-negative group. The differences between the groups, namely, NLR, PLR, and PNI, were compared. A sub-analysis compared preoperative and postoperative lymphocyte rates and NLR in groups with and without intraoperative complications. In addition, the postoperative mortality of the patients was followed up from our hospital's archive system and the Republic of Turkey’s Ministry of Health database.

Statistical analysis

IBM SPSS Statistics for Windows, version 26 (released 2019; IBM Corp., Armonk, New York, United States) was used to perform statistical analysis. Quantitative variables were expressed as mean ± standard deviation (SD), median, and minimum-maximum. Qualitative variables were reported as numbers and percentages. Kolmogorov-Smirnov and Shapiro-Wilk tests were used to evaluate the normality distribution. Independent-samples t-test and Mann-Whitney U test were used to compare groups according to normality test results. Qualitative variables were compared with chi-square tests (Pearson's chi-square and likelihood ratio test). P values <0.05 were considered statistically significant.

## Results

Of the 172 patients in the study, 96 (55.8%) were female. The mean age of all patients was 48.51±15.57 (18-90). The most common lesion location was the right lobe (68.6%). Right hepatectomy was performed in 58 (33.7%) patients, left hepatectomy in 11 (6.4%) patients, non-anatomical resection in 49 (28.5%) patients, and liver transplantation in 54 (31.4%) patients. Perioperative complications were seen in 30 (17.4%) patients: vascular injury in 12 (7%) patients and diaphragm injury in 18 (10.5%) patients. Of the patients who developed diaphragmatic lacerations, 11 were treated with prolene mesh placement, and seven were treated with primary repair. The patients with vascular injury were treated with pericardial grafts placed in the inferior vena cava and one in the portal vein. In the other seven patients, vascular injuries were treated with primary repair. The clinical parameters of the patients are shown in Table 1.

**Table 1:** Clinicopathological variables of the patients. SD: standard deviation, WBC: white blood cell, INR: international normalized ratio, NLR: neutrophil-to-lymphocyte ratio, PLR: platelet-to-lymphocyte ratio, PNI: preoperative nutritional index

Parameters	n (%) or value (range)
Age (mean±sd)	48.51±15.57 (18-90)
Gender	
Male	76 (44.2)
Female	96 (55.8)
Laboratory parameters (the day before surgery) (mean±SD)	
WBC (10^3^/mm^3^)	10.54±5.64 (2.5-46.9)
Neutrophil (%)	73.20±14.15 (34.4-97.9)
Lymphocyte (%)	17.52±10.66 (0.7-46.5)
Platelet (10^3^/mm^3^)	266.87±114.07 (24-735)
Eosinophil (%)	2.15±3.32 (0-26.2)
Albumin (g/dL)	3.29±0.77 (1.3-5.5)
INR	1.38±0.45 (0.85-3.76)
Creatinine (mg/dL)	0.8±0.5 (0.2-4.2)
NLR	9.01±13.81 (0.7-132.9)
PLR	25.11±27.35 (1.36-212.31)
PNI	41.00±11.62 (16.80-122.46)
Laboratory parameters (postoperative first day) (mean±sd)	
WBC	12.44±5.74 (0.01-28.6)
Neu (%)	81.30±11.58 (23-97.5)
Lym (%)	10.98±9.53 (0.40-74.30)
Platelet	231.19±107.19 (19-610)
Eosinophil	0.71±1.40 (0-11.5)
Albumin	2.99±0.55 (0.5-3.2)
INR	1.52±0.51 (0.5-3.2)
Creatinine	0.91±0.91 (0.3-9)
NLR	17.52±30.69 (1.21-240.75)
PLR	48.17±119.17 (1.58-1085)
PNI	35.63±6.94 (13.12-62.05)
Localization	
Right lobe	118 (68.6)
Left lobe	26 (15.1)
Both	28 (16.3)
Surgery type	
Right hepatectomy	58 (33.7)
Left hepatectomy	11 (6.4)
Non-anatomic resection	49 (28.5)
Liver transplantation	54 (31.4)
Intraoperative complication	
Yes	30 (17.4)
Vascular	12 (7)
Diaphragmatic	18 (10.5)
No	142 (82.6)
Morbidity	
Yes	49 (28.5)
No	123 (71.5)
Mortality	
Yes	33 (19.2)
No	139 (80.8)

The morbidity and mortality rates of the study were 28.5% and 19.2%, respectively. The most common postoperative complications were bile leakage (9.9%) and intra-abdominal abscess (8.7%). Postoperative complications and their treatments are shown in Table 2.

**Table 2:** Postoperative complications and their treatments.

Postoperative complications	Treatment	
Bile leakage (n=17)	Re-surgery (n=10)	Hepaticojejunostomy (n=7)
		Biliary tract revision (n=3)
	Spontaneous closure (n=7)	
An intra-abdominal abscess (n=15)	Re-surgery (n=2)	Abscess drainage
	Percutaneous intervention (n=13)	Abscess drainage
Pleural effusion (n=11)	Chest tube insertion	
Bleeding (n=3)	Re-surgery	
Acute rejection (n=3)	Re-transplantation	

Age, gender of patients, and preoperative laboratory parameters, including NLR, PLR, and PNI, were not different between the morbidity groups. However, the presence of perioperative vascular injury (P=0.040) and complications (P=0.047), low postoperative lymphocyte rates (P=0.038), and high postoperative NLR were associated with increased morbidity. In addition, the mortality rate was significantly increased in patients with morbidity (P<0.001). Table 3 shows the relationship of clinical factors to morbidity.

**Table 3:** Comparison of the morbidity groups. WBC: white blood cell, INR: international normalized ratio, NLR: neutrophil-to-lymphocyte ratio, PLR: platelet-to-lymphocyte ratio, PNI: preoperative nutritional index ^a^ mean + standard deviation, ^b^ n (%), ^c^ mean rank. *Independent samples t-test, **Pearson's chi-square, ***Mann-Whitney U test, ****likelihood ratio test

Parameters	Morbidity positive (n=49)	Morbidity negative (n=123)	P-value
Age ^a^	46.53±14.47	49.30±15.98	0.294*
Gender ^b^			0.255**
Male	25 (32.9)	51 (67.1)	
Female	24 (25)	72 (75)	
Laboratory parameters (the day before surgery)			
WBC ^c^	84.65	87.24	0.759***
Neu (%) ^c^	82.98	87.90	0.558***
Lym (%) ^c^	84.73	87.20	0.769***
Platelet ^c^	78.08	89.85	0.162***
Eosinophil ^c^	81.73	88.40	0.427***
Albumin ^a^	3.30	3.28	0.920*
INR ^c^	97.83	81.99	0.060***
Creatinine ^c^	86.40	86.54	0.986***
NLR ^c^	87.40	86.14	0.881***
PLR ^c^	84.05	87.48	0.684***
PNI ^c^	86.36	86.56	0.981***
Laboratory parameters (postoperative first day)			
WBC ^c^	96.20	82.63	0.107***
Neu% ^c^	93.71	83.63	0.230***
Lym% ^c^	74.03	91.47	0.038***
Platelet ^c^	77.12	90.24	0.119***
Eosinophil ^c^	90.04	85.09	0.538***
Albumin ^c^	79.98	89.10	0.278***
INR ^c^	82.15	88.23	0.468***
Creatinine ^c^	77.69	90.01	0.141***
NLR ^c^	98.50	81.72	0.046***
PLR ^c^	93.16	83.85	0.268***
PNI ^c^	85.03	87.09	0.807***
Localization ^b^			0.406****
Right lobe	34 (28.8)	84 (71.2)	
Left lobe	5 (19.2)	21 (80.8)	
Both	10 (35.7)	18 (64.3)	
Surgery type ^b^			0.275****
Right hepatectomy	21 (36.2)	37 (63.8)	
Left hepatectomy	2 (18.2)	9 (81.8)	
Non-anatomic resection	10 (20.4)	39 (79.6)	
Liver transplantation	16 (29.6)	38 (70.4)	
Intraoperative complication ^b^			0.047**
Yes	13 (43.3)	17 (56.7)	
No	36 (25.4)	106 (74.6)	
Vascular complication ^b^			0.040****
Yes	7 (58.3)	5 (41.7)	
No	42 (26.3)	118 (73.8)	
Diaphragmatic complication ^b^			0.630**
Yes	6 (33.3)	12 (66.7)	
No	43 (27.9)	111 (72.1)	
Mortality ^b^			<0.001**
Yes	18 (54.5)	15 (45.5)	
No	31 (22.3)	108 (77.7)	

Because intraoperative complications affect morbidity, a sub-analysis was performed by comparing preoperative and postoperative lymphocyte rates and NLR in groups with and without intraoperative complications to rule out this situation. In this sub-analysis, no difference was found between the groups in both the preoperative and postoperative evaluated serum values. Table 4 compares the intraoperative complications groups according to the lymphocyte rates and NLRs.

**Table 4:** Comparison of the intraoperative complications groups according to lymphocyte counts and NLRs. NLR​​​​​​: neutrophil-to-lymphocyte ratio, ​^a^ mean rank, *Mann-Whitney U test

Parameters	Perioperative complication positive (n=30)	Perioperative complication negative (n=142)	P-value
Preoperative lymphocyte (%) ^a^	73.52	89.24	0.116*
Preoperative NLR ^a^	87.72	79.85	0.148*
Postoperative lymphocyte (%) ^a^	72.42	89.48	0.088*
Postoperative NLR ^a^	99.70	83.71	0.110*

## Discussion

At present, more studies have shown that inflammation plays a vital role in disease development, occurrence, and metastasis and affects the host’s immune regulation [9]. The present study investigated the significance of the NLR, PLR, and PNI as predictors of morbidity in liver resection patients for alveolar echinococcosis. Our results show that preoperative NLR, PLR, and PNI levels did not affect morbidity. However, postoperative NLR levels were significantly higher in patients with postoperative morbidity, whereas postoperative PLR and PNI levels were similar between the morbidity groups. In addition, low lymphocyte rates were associated with increased morbidity. To the best of our knowledge, this is the first study to evaluate the effect of these three inflammatory markers (preoperatively and postoperatively) on morbidity in patients operated on for hepatic alveolar echinococcosis.

Alveolar echinococcus, responsible for 3% of all echinococcal lesions in the liver, is common in Central and Western Europe, North America, China, Turkey, and Iran [2]. The incubation period is between five and 15 years. This prolonged incubation period delays the diagnosis of the cases and complicates the surgery [4]. The most effective treatment is resection of the mass with a clear surgical margin. However, liver transplantation can be performed as a curative method in patients who cannot be treated with resection [10]. All complications after the operation prolong the patient's hospitalization period, increase the financial burden, and reduce the patient's comfort. It is essential to determine the complication factors to prevent such problems. In the present study, perioperative and vascular complications were found to increase morbidity. In addition, as morbidity increased, mortality also increased.

Relationships between preoperative nutritional and immunological status and the postoperative period have been investigated in various diseases. Many studies have reported that the nutritional and immunological statuses affect postoperative outcomes [11]. Alveolar echinococcal disease is also a disease with severe inflammation, and it is known that the inflammatory process plays a vital role in all its stages [12]. Many parameters related to preoperative prognostic indicators have been studied, but the high cost, lack of standardization, and unsuitability for widespread use of these parameters have limited their clinical use. Complete blood count and biochemical evaluation are the primary laboratory parameters. These simple tests can determine the nutritional status because they are inexpensive and studied in all patients. The present study determined basic hematological parameters (neutrophil count, lymphocyte count, and platelet count) and serum albumin levels, and the NLR, PLR and PNI values were calculated. Postoperative lower lymphocyte rates and higher NLR levels were significantly associated with increased morbidity rates.

The first clinical study of NLR in humans found that NLR was a valuable marker for acute appendicitis. Later, it was shown that NLR could be used as a cancer biomarker and an important inflammatory parameter for various diseases, such as incarcerated hernias and acute appendicitis [13-15]. Meanwhile, PLR is a newly discovered marker, and its first use was in periampullary tumors [16]. PLR has been helpful in many fields over time [11]. First proposed by Buzby et al., the PNI is calculated mainly by counting serum albumin and peripheral blood lymphocytes and can comprehensively reflect patients' nutritional and immune status [17]. Ren et al.’s study on alveolar echinococcus with 242 patients showed that the five-year overall survival rate for the low-PNI group was lower than that for the high-PNI group (37.7% vs. 71.6%; P<0.001). In the same study, the five-year overall survival rate in the low-PLR group was higher than that in the high-PLR group (70.4% vs. 24.3%; P<0.001), and the five-year overall survival rate in the low-NLR group was higher than that in high-NLR group (67.2% vs. 28.8%; P<0.001) [18]. As there is no study on the morbidity factors of alveolar hydatid cysts, our study dramatically contributes to the literature.

Limitations and suggestions

Our clinical study has some limitations. First, this is a small sample retrospective study, and there may be selection bias in the data collection process. Second, this study did not include an evaluation of other inflammatory markers, such as C-reactive protein, interleukin, and procalcitonin. Studies with more patients and different inflammatory parameters are needed to investigate the factors affecting morbidity. In addition, there is a need for new studies that will determine the factors affecting mortality and inflammatory parameters according to lesion localization (e.g., right lobe vs. left lobe) and the type of surgery (e.g., hepatectomy vs. liver transplantation).

## Conclusions

Alveolar echinococcosis is a parasitic infection that mainly spreads to the liver, has a slow course, is diagnosed late, and can be encountered with metastatic lesions, such as malignancy. Delayed diagnosis both complicates the surgery and increases morbidity and mortality. This study compared three basic inflammatory rates/formulas, contrary to what was thought that preoperative parameters did not affect morbidity. At the same time, the increase in postoperative NLR levels and the decrease in lymphocyte rates increased morbidity. In addition, intraoperative complications (primarily vascular complications) increased morbidity, and mortality increased as morbidity increased.
